# Iron-modified activated carbon derived from agro-waste for enhanced dye removal from aqueous solutions

**DOI:** 10.1016/j.heliyon.2021.e07191

**Published:** 2021-05-29

**Authors:** Fateme Barjasteh-Askari, Mojtaba Davoudi, Maryam Dolatabadi, Saeid Ahmadzadeh

**Affiliations:** a Department of Environmental Health Engineering, School of Health, Torbat Heydariyeh University of Medical Sciences, Torbat Heydariyeh, Iran; b Health Sciences Research Center, Torbat Heydariyeh University of Medical Sciences, Torbat Heydariyeh, Iran; c Department of Environmental Health Engineering, School of Public Health, Tehran University of Medical Sciences, Tehran, Iran; d Department of Environmental Health Engineering, School of Health, Mashhad University of Medical Sciences, Mashhad, Iran; e Social Determinants of Health Research Center, Mashhad University of Medical Sciences, Mashhad, Iran; f Environmental Science and Technology Research Center, Department of Environmental Health Engineering, Shahid Sadoughi University of Medical Sciences, Yazd, Iran; g Pharmaceutics Research Center, Institute of Neuropharmacology, Kerman University of Medical Sciences, Kerman, Iran; h Pharmaceutical Sciences and Cosmetic Products Research Center, Kerman University of Medical Sciences, Kerman, Iran

**Keywords:** Activated carbon, Adsorption, Agro-waste, Dye removal, Iron modification, Pistachio shell

## Abstract

**Background and aim:**

Finding a cost-effective adsorbent can be an obstacle to large-scale applications of adsorption. This study used an efficient activated carbon adsorbent based on agro-waste for dye removal.

**Methods:**

Pistachio shells as abundant local agro-wastes were used to prepare activated carbon. Then, it was modified with iron to improve its characteristics. Acid red 14 was used as a model dye in various conditions of adsorption (AR14 concentration 20–150 mg L^−1^, pH 3–10, adsorbent dosage 0.1–0.3 g L^−1^, and contact time 5–60 min).

**Results:**

A mesoporous adsorbent was prepared from pistachio shells with 811.57 m^2^ g^−1^ surface area and 0.654 cm^3^ g^−1^ pore volume. Iron modification enhanced the characteristics of activated carbon (surface area by 33.3% and pore volume by 64.1%). Adsorption experiments showed the high effectiveness of iron-modified activated carbon for AR14 removal (>99%, >516 mg g^−1^). The adsorption followed the pseudo-second kinetic model (k = 0.0005 g mg^−1^ min^−1^) and the Freundlich isotherm model (K_f_ = 152.87, n = 4.61). Besides, the reaction occurred spontaneously (ΔG^0^ = −36.65 to −41.12 kJ mol^−1^) and was exothermic (ΔH^0^ = −41.86 kJ mol^−1^ and ΔS^0^ = −3.34 J mol^−1^ K^−1^).

**Conclusion:**

Iron-modified activated carbon derived from pistachio shells could be cost-effective for the treatment of industrial wastewater containing dyes.

## 1 Introduction

Adsorption is a widely used technique for the removal of pollutants from aqueous solutions. This process entails the mass transfer of solutes from the bulk solution to the adsorbent surface. This technique has many advantages over other technologies for the removal of pollutants, including the ease of design and operation, low capital cost, flexibility, and high effectiveness without producing secondary pollutants [1]. Adsorbents are efficient in uptaking a wide variety of pollutants ranging from organic matters such as dyes [2] to inorganics such as heavy metals [3].

Activated carbon has been shown to be an efficient adsorbent in various studies, as it provides a very large specific surface area, high porosity, and good affinity to various types of pollutants [4]. The pores in the activated carbon structure can be found in various sizes, ranging from micropores (<2 nm) to mesopores (2–50 nm) and macropores (>50 nm) [5]. Activated carbon is produced from the processing of materials that are rich in carbon, including bamboo, wood, coal, lignin, etc. Using agro-waste such as pistachio shells as the precursor for activated carbon may increase the sustainability of the process and decrease production costs.

The characteristics of activated carbon depend largely on the fabrication method. Activated carbon is produced with physical or chemical processes. In the physical method, the material is paralyzed by heating at high temperatures in the presence of inert gas and then is activated in a hot oxidizing atmosphere. In the chemical method, the raw material is first immersed in a strong acid, base, or salt solution and then heated at relatively low temperatures. Chemical preparation methods are preferred over physical methods as they decrease the preparation costs, because of relatively low temperatures applied, and produce an adsorbent with better characteristics, as shown in the literature [6].

The intrinsic characteristics of pure activated carbon are not sometimes adequate to remove the pollutants favorably. Therefore, activated carbon may undergo modification processes. The modification of activated carbon is done for various purposes, including enhancing its sorption capacity by incorporating nanomaterials [7], promoting its separation properties by impregnation with magnetic particles [8], and increasing its disinfection characteristics by loading with antimicrobial agents such as silver [9]. In this vein, iron has been used for the modification of various adsorbents including biochars [10], zeolites [11], montmorillonite [12], and activated carbon [13]. Iron oxide nanoparticles, per se, are efficient adsorbents that can be used alone or in combination with other adsorbents to remove a wide variety of pollutants including textile dyes [14]. When activated carbon is added to a precursor solution containing iron ions to produce iron-activated carbon composites, the ions can diffuse deeply into the internal pores of activated carbon and link with its surface functional groups, implying an efficient impregnation [13]. Using iron-oxide-decorated activated carbon as an adsorbent may benefit from the advantages of both materials.

The current study aimed to synthesize a cost-effective adsorbent made from agro-waste for the removal of a model textile dye (Acid red 14) from aqueous solutions. The pistachio shell was used as the precursor for activated carbon, as this type of agro-waste is largely produced in our country, Iran [15]. The pistachio shell-derived activated carbon was modified with iron to enhance its adsorptive characteristics for AR14 removal. Finally, a series of kinetic, isotherm and thermodynamic studies were conducted.

## 2 Materials and methods

### 2.1 Chemicals

A regional textile factory kindly supplied Acid Red 14 (AR14, azo dye, ≥98% purity) and we used it without extra purification. Other chemicals were the products of Merck (Germany), including NaOH (≥97% purity), HCl (37% purity), NaNO_3_ (65% purity), H_3_PO_4_ (85% purity), FeCl_3_ (99.9% purity), and KNO_3_ (99% purity).

### 2.2 Adsorbent preparation

For the preparation of pistachio shell-derived activated carbon, a method described by Enaime, Ennaciri [6] was followed, with slight modifications. First, pistachio shells were washed thoroughly with water, then ground, and dried in an oven at 110 ± 5 °C for 1 h. The dried powders were mixed with concentrated phosphoric acid at a ratio of 1–10 (w/w) for 3 h at room temperature on a magnetic stirrer. The mixture was filtered using a paper filter and the separated mass was heated in a furnace. To produce activated carbon, a temperature program was set with a gradual increase to 900 °C in 3 h, maintaining the temperature for 1 h, and a gradual decrease to the initial temperature in 3 h. After being washed with Doubled Distilled Water (DDW) to neutral pH (6.5–7.5), the activated carbon product was dried at 110 ± 5 °C and sized with a 20-mesh (850 μm) sieve.

For the preparation of iron-modified-activated carbon, 10 g of the as-synthesized activated carbon powders were mixed with 200 mL of 36% nitric acid and kept for 1 h at 80 °C. Then, the separated mass remained in the room temperature for 24 h to be air-dried. For iron modification, 10 g of the dried powders were mixed with 200 mL of 3 M FeCl_3_ for 3 h at room temperature. After passing through a paper filter, the remaining mass was dried for 1 h at 110 ± 5 °C, followed by heating at 750 ± 5 °C for 1 h under a nitrogen stream. After cooling down, the iron-modified product was washed with DDW, dried for 1 h at 110 ± 5 °C, and sized with a 20-mesh sieve.

### 2.3 Adsorbent characterization

The Brunauer–Emmett–Teller (BET) analysis was conducted using a surface analyzer (BELSORP-mini II, BEL Inc., Japan) to evaluate the surface characteristics of adsorbents. In this analysis, N_2_ adsorption/desorption isotherms were investigated at the temperature of 77 K. The manufacturer's software calculated the BET specific surface area and total pore volume of adsorbents. The external surface area (S_ext_), the micropore volume (V_mic_), and mean pore diameter (D_p_) were calculated by the t-plot method. A field emission scanning electron microscope coupled with an energy-dispersive X-ray system (FESEM-EDS; MIRA III model, Tescan, Czech Republic) was used to determine the morphological characteristics and the elemental composition of the materials. The point of zero charge (pH_pzc_) of the Fe-modified activated carbon was determined following the Doltabadi, Alidadi [16] method using a pH meter apparatus (HACH HQ440D multi).

### 2.4 Experimental design

The experimental studies were carried out to determine the effectiveness of iron-modified activated carbon as the adsorbent for the removal of AR14 as a model dye. For modeling the process, a Central Composite Design (CCD) under Response Surface Methodology (RSM) in Design Expert 7 software® was employed. The iron-modified activated carbon was used for dye adsorption, as the characterization studies and a pilot study showed its superiority over unmodified activated carbon. Table 1 shows the factors and their levels in the CCD.

**Table 1:** Coded and real values of independent factors used in CCD.

Coded Variables (X_i_)	Factors (U_i_)	Experimental Field				
		−α	−1	0	+1	+α
X_1_	A: Initial AR14 concentration (mg L^−1^)	20	52.5	85.0	117.5	150
X_2_	B: Solution pH	3.00	4.75	6.50	8.25	10.00
X_3_	C: Adsorbent dosage (g L^−1^)	0.10	0.15	0.20	0.25	0.30
X_4_	D: Contact time (min)	5.00	18.75	32.50	46.25	60.00

The experimental data were sought to fit a second-order polynomial model expressed as Eq. (1) [17]:

(1) $$Y=\beta_{0}+\overset{n}{\underset{i=1}{\sum }}\beta_{i}X_{i}+\overset{n}{\underset{i=1}{\sum }}\beta_{ii}X_{i}^{2}+\overset{n-1}{\underset{i=1}{\sum }}\overset{n}{\underset{j=i+1}{\sum }}\beta_{ij}X_{i}X_{j}$$

In this equation, *Y* denotes the predicted AR14 removal percentage, *β*_*0*_ the intercept parameter, *β*_*i*_ the linear coefficients, *β*_*ii*_ the quadratic coefficients, *β*_*ij*_ the interaction coefficients, and *X*_*i*_, *X*_*j*_ the coded values of independent variables.

### 2.5 Adsorption studies

First, a series of experiments were carried out to confirm the superiority of iron-modified activated carbon over unmodified activated carbon for the removal of AR14. The next experiments were conducted using the superior adsorbent according to the CCD to optimize the condition for AR14 removal. To do the experiments, a glass vessel was filled with 50 mL of the solution containing the given concentration of the model dye and the pH was adjusted (with dilute HCl or NaOH). After adding a predetermined amount of the adsorbent, the suspension was mixed at 100 rpm on a magnetic stirrer. At the predetermined contact time, the supernatant was analyzed for the residual dye after centrifugation at 1,000 rpm for 5 min.

Finally, kinetic, isotherm, and thermodynamic studies were performed to determine the adsorption reaction characteristics. The kinetic studies were carried out at an initial dye concentration of 100.00 mg L^−1^, adsorbent dose of 0.25 g L^−1^, solution pH of 4.50, and contact time of 0–40.00 min. Adsorption isotherms were investigated at initial dye concentrations of 30–150 mg L^−1^, adsorbent dose of 0.25 g L^−1^, solution pH of 4.50, and equilibrium contact time of 40.00 min. For the thermodynamic studies, the experimental conditions were initial dye concentrations of 50–150 mg L^−1^, solution pH of 4.50, adsorbent dose of 0.25 g L^−1^, solution temperatures of 278–338 K, and contact time of 40.0 min.

### 2.6 Chemical and statistical analysis

Residual AR14 in the reactor effluent was measured using a UV-vis spectrometer (T80 + UV/VIS) at a wavelength of 515 nm. The measurements were used to calculate the removal percentage of the dye using Eq. (2):

(2) $$\text{Dye removal }\left(\text{\%}\right)=\frac{{\text{C}}_{0}-{\text{C}}_{\text{t}}}{{\text{C}}_{0}}\times 100$$

In this equation, *C*_*0*_ (mg L^−1^) is the initial dye concentration and *C*_*t*_ (mg L^−1^) is the concentrations at time *t.* The day amount uptaken by the adsorbent at equilibrium, *qe* (mg g^−1^), was quantified using Eq. (3):

(3) $${\text{q}}_{\text{e}}=\frac{\left({\text{C}}_{0}-{\text{C}}_{\text{e}}\right)\times \text{V}}{\text{W}}$$

In the above equation, *C*_*e*_ is the equilibrium dye concentration (mg L^−1^), *V* denotes the solution volume (L), and *W* indicates the dry adsorbent mass used in the solution (g).

The statistical models were developed and evaluated by the Analysis of Variance (ANOVA) at a significance level of 95% [18]. The goodness of fit of the regression model was expressed by R-square (R^2^), adjusted R-square (Adj. R^2^), and predicted R-square (pred. R^2^). OrigingPro9 software® was employed to fit the experimental data to the non-linear forms of kinetic and isotherm model equations at a significance level of 95%. The error functions including Adj. R^2^, the Sum of Squared Errors (SSE), and Chi-square (χ^2^) were employed to determine the adequacy of these models. The models were finally judged by performing the Akaike Information Criterion (AIC) test.

## 3 Results and discussion

### 3.1 Characterization

**Figure 1:**
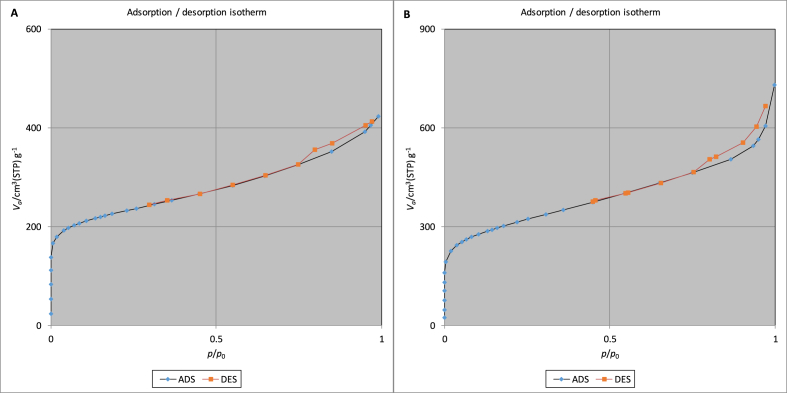
Adsorption-desorption isotherms of A) activated carbon and B) iron-modified activated carbon.

Adsorption-desorption and BET analyses were first used to specify iron-modified and unmodified activated carbon. As shown in Figure 1, hysteresis is observed in both curves of adsorption-desorption. Based on the classification of the International Union of Pure and Applied Chemistry (IUPAC), both curves are closest in shape to type IV physisorption isotherms [19]. The IUPAC classification is based on the strength of the adsorbent surface-adsorptive interaction and the absence or presence of pores. Type IV physisorption isotherms indicate mesoporous adsorbents with pore diameters of 2–50 nm, contributing to relatively strong adsorption. The hysteresis loops observed in the curves are of type H3 loops, which are recognized by an adsorption branch resembling a type II isotherm [20]. The presence of the H3 loop indicates micropores and/or mesopores with ink bottle shapes [21]. Thus, one can infer that the isotherm is a mixture of type II and IV. Mixed types of adsorption isotherms may be observed in some adsorption systems. The sharp curvatures at the beginning of the plots represent the completion of monolayer coverage and the onset of multilayer adsorption, which, in this case, occurs at very low p/p_0_ values [20].

Based on the findings of physisorption isotherms, the Brunauer–Emmett–Teller (BET) method was used to further evaluate the surface areas of adsorbents. As can be seen in Table 2, surface modification with iron enhanced the characteristics of activated carbon and increased the BET specific surface area by 33.3% and the total pore volume by 64.1%. These values have been reported as 17.5% and 24.1% increases, respectively, by Liu, Zhang [22] after the modification of activated carbon prepared from Trapa natans husk with iron. This implies that iron impregnation did not block carbon pores. Based on the results of the t-plot method in Table 2, the external surface areas (a_2_) comprised only 5.92% and 7.73% of the total surface areas of unmodified and modified adsorbents. Therefore, one can conclude that the adsorbents were mesoporous with large internal surface areas.

**Table 2:** The BET and t-plot results for evaluating the surface characteristics of adsorbents under STP (T = 273.15 K, P = 101.3 kPa).

Analysis	Parameter	Description	Unit of expression	Activated carbon	Iron-modified activated carbon
BET	V_m_	Monolayer volume	cm^3^ g^−1^	186.46	248.72
	a_s,BET_	BET specific surface area	m^2^ g^−1^	811.57	1082.5
	C	Energy constant of the first layer	-	11268	1162.4
	V_p_	Total pore volume	cm^3^ g^−1^	0.654	1.0736
t-plot	a_1_	Total specific surface area	m^2^ g^−1^	991.41	1196.2
	a_2_	External surface area	m^2^ g^−1^	58.67	88.22
	V_2_	Pore volume	cm^3^ g^−1^	0.5048	0.7039
	2t	Pore diameter	nm	1.0758	1.2629

**Figure 2:**
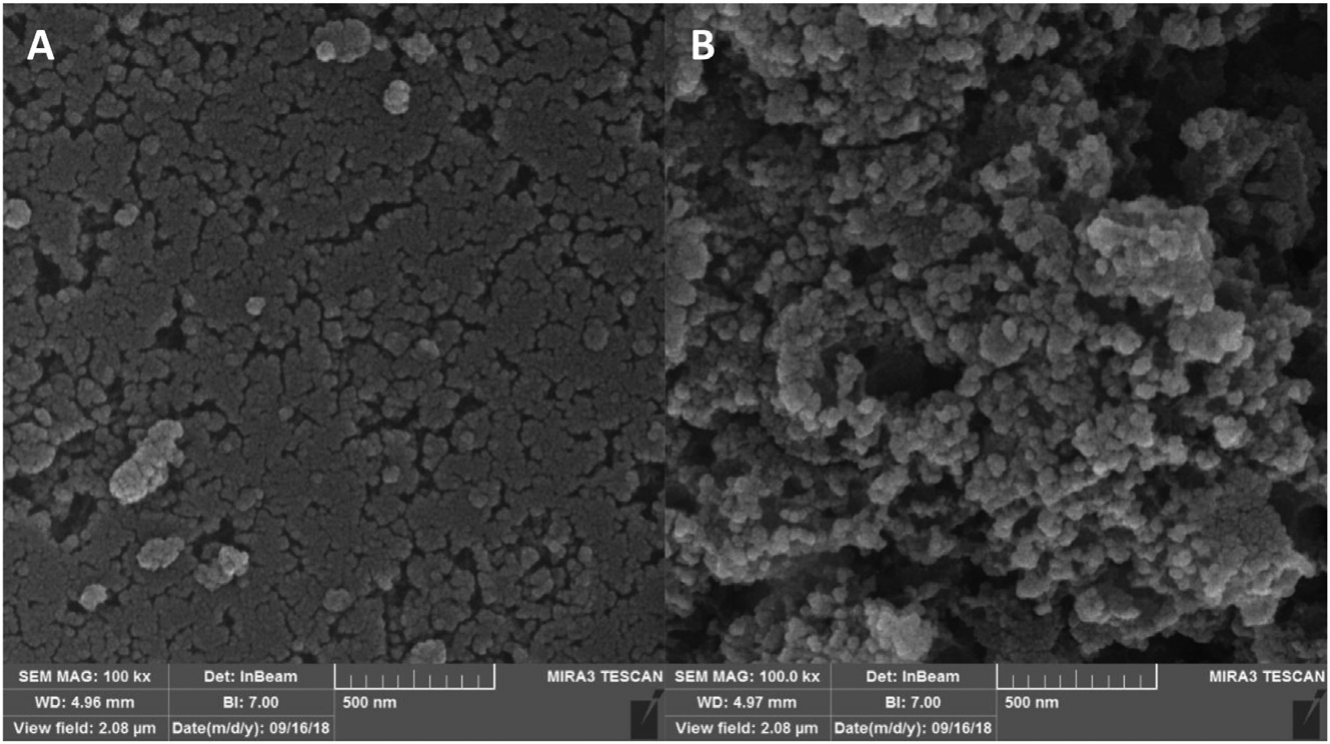
The FE-SEM images of A) activated carbon and B) iron-modified activated carbon.

**Figure 3:**
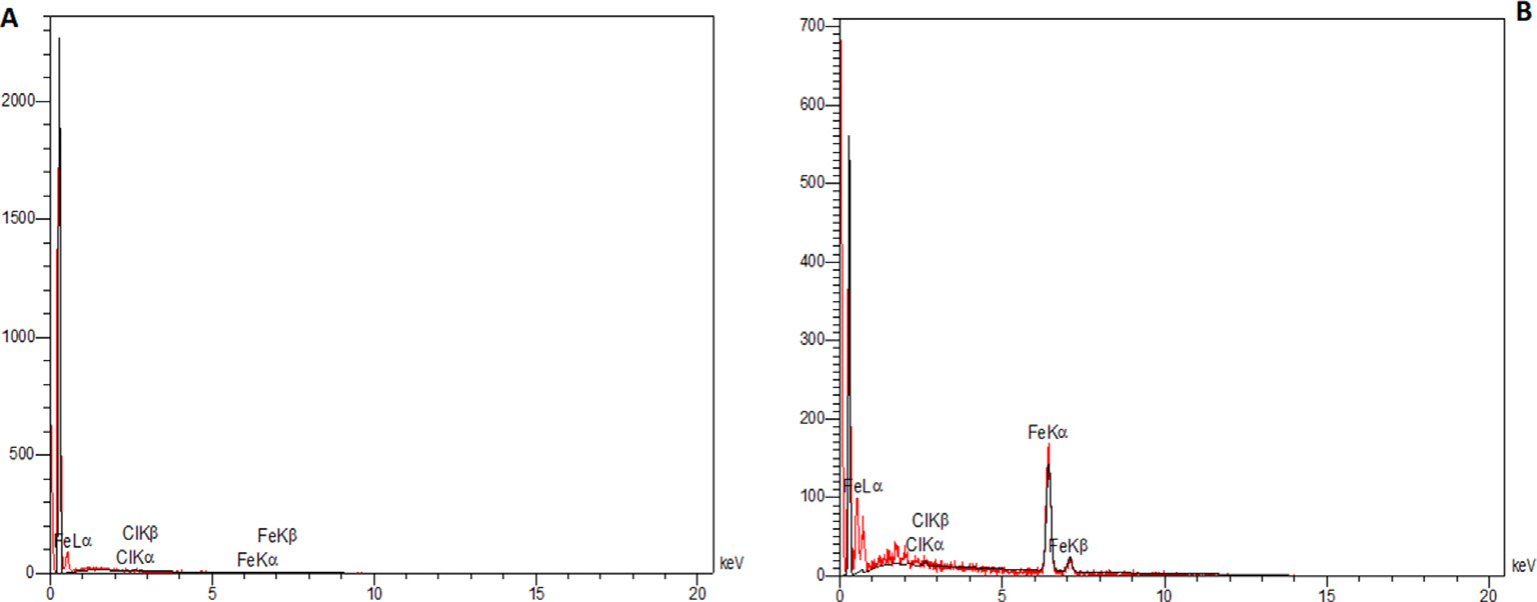
The EDX spectra of A) activated carbon and B) iron-modified activated carbon.

The surface morphology of adsorbents obtained using FESEM is depicted in Figure 2. As can be seen, activated carbon had a fine, even surface with low surface porosity. However, after modification with iron, a rough surface with more porosity appeared. The EDX analysis (Figure 3) showed that the as-prepared activated carbon comprised 99.58% wt. carbon. However, after modification with FeCl_3_, the composition was 71.49% wt. carbon, 28.33% wt. Fe, and 0.19% wt. Cl. These values show the successful iron impregnation of carbon particles and nearly complete oxidation of iron ions to iron oxides. Braun, Borba [23] used a combination of FeSO_4_ and FeCl_3_ for the impregnation of commercial activated carbon particles and showed the percentages of 46%, 32%, and 20% for carbon, iron, and oxygen, respectively. These results corroborate our findings, except that we did not measure the oxygen element in the composition.

### 3.2 Dye adsorption experiments

**Figure 4:**
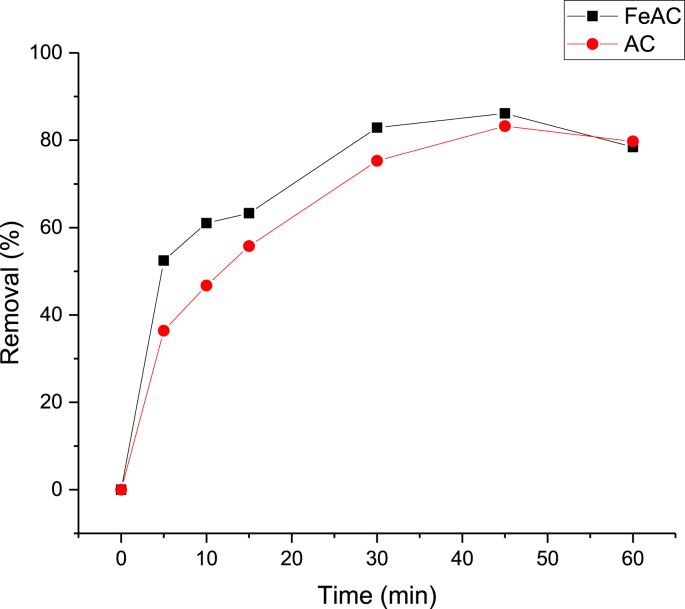
The comparative results of AR14 removal by activated carbon (AC) and iron-modified activated carbon (FeAC) [dye concentration 75.0 mg L^−1^, pH 7.0, adsorbent dose 0.2 g L^−1^].

A series of adsorption experiments were carried out to compare the efficacy of activated carbon and iron-modified activated carbon for AR14 removal from aqueous solutions. The results are depicted in Figure 4. As shown, iron modification enhanced the efficacy of activated carbon prepared from pistachio shells for the removal of AR14, especially at the initial contact times. This could be due to the improved characteristics of activated carbon following the modification, including enhanced specific surface areas and pore volumes. Due to its better efficacy, iron-modified activated carbon was used in further experiments.

The optimization experiments were conducted using iron-modified activated carbon for the AR14 removal from aqueous solutions. A summary of the results obtained under various experimental conditions defined by CCD is presented in Table S1 in the supplementary file. In brief, the removal efficiency was in the range of 35.34–99.99% with a mean ± SD of 69.57 ± 18.76%. A regression analysis was used to describe the relationship between the independent variables and the response, leading to a quadratic model expressed as Eq. (4):

(4) $$Y=83.57-11.34X_{1}-8.37X_{2}+9.22X_{3}+5.84X_{4}-2.22X_{1}X_{3}-2.10X_{1}X_{4}+2.58X_{2}X_{3}-6.30X_{2}^{2}-6.52X_{3}^{2}-4.67X_{4}^{2}$$

In this equation, *Y* is the removal rate of AR14 (%), and *X1* to *X4* represents the coded values of independent variables including initial dye concentration, initial solution pH, adsorbent dose, and contact time, respectively. The adequacy of the model was investigated using the ANOVA test. The results showed that the model was highly significant at a confidence level of 95% (F = 59.30; p < 0.01). The correlation coefficient, R^2^, between the predicted and actual values of dye removal was computed to be 0.9690, implying that only 3.1% of the total variance in the response could not be explained by the model. The difference between adjusted R^2^ and predicted R^2^ was less than 0.20 (0.9526 vs. 0.9090, respectively), indicating again the significance of the model. The value of adequate precision was 29.10, representing a desirable signal-to-noise ratio.

### 3.3 Effect of operational parameters

**Figure 5:**
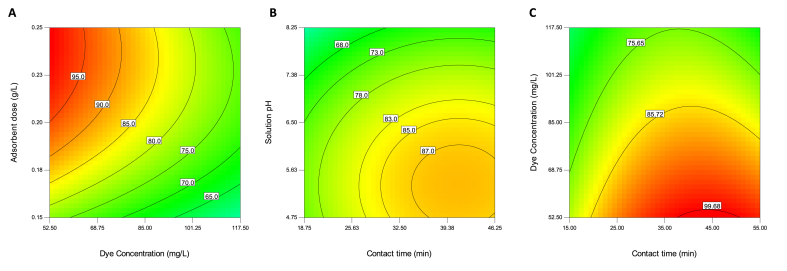
Removal efficiency of AR14 using iron-modified activated carbon as a function of A) adsorbent dose and dye concentration [solution pH 6.5; contact time 32.5 min], B) solution pH and contact time [dye concentration 85.0 mg L^−1^; adsorbent dose 0.2 g L^−1^], and C) dye concentration and contact time [solution pH 6.5; adsorbent dose 0.2 g L^−1^].

It is well known in the adsorption systems that the removal rate is correlated reversely with the initial dye concentration [3, 24] and directly with the adsorbent dose [25, 26]. This behavior was also established in the current work. For example, as shown in Figure 5A, at an adsorbent dose of 0.15 g L^−1^, the AR14 removal rate decreases from 77.25 to 59.06% when the initial dye concentration increases from 52.50 to 117.50 mg L^−1^, and at an initial dye concentration of 52.50 mg L^−1^, the removal percentage increases from 77.25 to about 99.77% by increasing adsorbent dose from 0.15 to 0.25 g L^−1^. The figure also shows that the removal efficiencies of above 95% are always obtained at initial dye concentrations ≤65.0 mg L^−1^ and adsorbent doses ≥0.2 g L^−1^. Generally, it should be noted that the higher the ratio of adsorbent to adsorbate, the higher the removal rate of the adsorbate. This ratio is increased by decreasing the adsorbate concentration and/or increasing the adsorbent dose.

The interactive effect of solution pH and contact time is observed in Figure 5B. As can be seen, increasing pH leads to a decreasing removal percentage of AR14. For example, at a contact time of 18.75 min, the removal efficiency decreases from 75.35 to 58.58% when the solution pH increases from 4.75 to 8.25. Moreover, removal percentages of above 85.00% are obtained when contact time is above 30.0 min and solution pH is below 6.60. Studies dealing with the adsorptive removal of AR14 also observed an increased removal rate at lower pH values. For example, Arami, Limaee [27] and Khodam, Rezvani [28] reported solution pH values as low as 2 and 3.5 to be optimal for the removal of AR14 using the eggshell membrane biosorbent and the multi-walled carbon nanotubes-based adsorbent, respectively. For explaining the results obtained in the current study, it is worthy to note the pH_pzc_ of the adsorbent. We determined pH_pzc_ to be 6.15, implying that the surface of the adsorbent was positive at pH values of lower than 6.15, neutral at pH of 6.15, and negative at pH values of higher than 6.15. Therefore, one can conclude that anionic dye AR14 is adsorbed most efficiently at acidic solutions due to the electrostatic attraction between anionic dye molecules and positively charged adsorbent surfaces. Overall, any pH value lower than 6.15 can be optimal for the removal of AR14 using the as-prepared adsorbent. We selected pH 4.50 for kinetic, isotherm, and thermodynamic experiments.

In the case of contact time, Figure 5B shows that at a solution pH of 4.75, increasing contact time from 18.75 to 46.25 min leads to increasing removal efficiency from 75.35 to 86.99%. This trend is in line with the results of previous studies [29, 30]. Another finding of the study was that the equilibrium time depended on the initial dye concentration. Figure 5C shows the trend of removal rate as a function of contact time and initial dye concentration. As can be seen, the equilibrium times were 43.68, 40.23, and 37.71 min at initial AR14 concentrations of 54.38, 90.60, and 117.08 mg L^−1^, respectively. The corresponding removal rates were 99.68%, 85.72%, and 75.65% and the corresponding adsorption capacities were 271.03, 388.31, and 442.85 mg g^−1^, respectively. In the literature, Khodam, Rezvani [28] investigated the removal rate of AR14 in the presence of a multi-walled carbon nanotube adsorbent in a range of contact time from 30 to 90 min. They reported the optimum contact time of 60 min; however, their study was not designed to assess the interaction between initial dye concentration and the equilibrium time. In another study, Venckatesh, Amudha [31] reported the concentration-dependent equilibrium time in an adsorption system containing direct red 28 and carbon adsorbent (prepared from Pomegranate rind) but the trend was not reported. Ozcan, Tor [32] demonstrated an ascending trend for the relationship between equilibrium time and initial aldrin concentration in the presence of montmorillonite adsorbent (60 min at 5 μg L^−1^ to 120 min at 10–20 μg L^−1^), which is in contrast to the findings of the present study. The descending trend for the equilibrium time as a function of initial AR14 concentration in this study may be attributed to the more rapid occupation of adsorption sites by adsorbate molecules when higher concentrations of adsorbate are applied. As also observed in the current study, after reaching the equilibrium time, desorption occurs and the removal rate of dye decreases. In further experiments, the equilibrium time was set at 40 min.

### 3.4 Kinetic studies

Pseudo-first-order and pseudo-second-order models were employed to explain the adsorption reactions. The model equations, parameters, and descriptions are presented in Table S2 in the supplementary file. The obtained experimental data were fitted to both kinetic models and the outcomes are depicted in Figure S1 in the supplemental file. A summary of the results is presented in Table 3. Based on the statistics, the pseudo-second-order model was better than the pseudo-first-order model for describing the experiments (Adj. R^2^ = 0.9614; AIC = 54.26). It means that the mechanism of AR14 uptake onto the adsorbent was chemisorption.

**Table 3:** The kinetics parameters and statistical data of AR14 removal using iron-modified activated carbon.

Kinetic model	q_e_	k	Statistics			ANOVA		AIC
			Reduced χ^2^	SSE	Adj. R^2^	F-value	Prob > F	
Pseudo-first order	277.98	0.151 (min^−1^)	631.79	3158.96	0.9446	238.39	<0.01	56.78
Pseudo-second order	332.98	0.0005 (g mg^−1^ min^−1^)	440.61	2203.03	0.9614	342.91	<0.01	54.26

### 3.5 Isotherm studies

The Langmuir, Freundlich, Jovanovich, Redlich-Peterson, and BET isotherm models were used to examine the adsorption system at equilibrium. The model equations, parameters, and descriptions are presented in Table S3 in the supplementary file. The obtained isotherm plots (Figure S2) were used to calculate the isotherm parameters, as listed in Table 4. Statistical indices were used to identify the most adequate model describing the adsorption at equilibrium. The AIC results indicated the order of the adequacy of the models as follows: Freundlich > Langmuir > Jovanovic > BET > Redlich-Peterson. As the Freundlich isotherm best fitted the data, the following is inferred: 1) The sorption sites of the adsorbent were heterogeneous, 2) The adsorption was multilayer, 3) The sorption sites had different energies of adsorption, and 4) The AR14 molecules non-uniformly distributed to the binding sites of the adsorbent. The *n* and *K*_*f*_ values in the Freundlich model are used to determine the desirability of adsorption [33]. A greater *n* value (in the range of 1–10) and a greater *K*_*f*_ value are in favor of stronger adsorption. The closer the *n* value to 10, the more heterogeneous the adsorbent surface. Thus, the heterogeneity of the adsorbent was moderate (n = 4.61).

**Table 4:** The isotherm parameters and statistical data of AR14 removal using iron-modified activated carbon.

Isotherm	Outputs				
	Constants	Reduced χ^2^	SSE	Adj. R^2^	AIC
Langmuir	b = 0.82 L mg^−1^	1419.20	5676.75	0.9200	59.11
	q_m_ = 328.44 mg g^−1^				
Freundlich	K_f_ = 152.87 (mg g^−1^) (mg^−1^)^1/n^	148.11	592.44	0.9916	45.55
	n = 4.61				
Jovanovic	q_m_ = 307.10 mg g^−1^	2412.24	9648.97	0.8640	62.30
	K_j_ = ‒0.61 L mg^−1^				
Redlich-Peterson	A = 4036.66 L g^−1^	191.69	575.08	0.9892	75.38
	B = 25.42 (mg L^−1^)^‒g^				
	g = 0.79				
BET	qs = 249.71 mg g^−1^	28.93	86.79	0.9984	64.03
	CBET = 300.64 L mg^−1^				
	Cs = 163.27 mg L^−1^				

### 3.6 Estimation of adsorption capacity

Adsorption capacity is an important criterion related to the uptake potential of a sorbent that justifies the economic aspects of using a special adsorbent for real-scale application. In this study, various adsorption capacities were calculated for AR14 sorption onto the adsorbent from isotherm models. They included 328.44 mg g^−1^ from the Langmuir isotherm model, 307.10 mg g^−1^ from the Jovanovic isotherm model, and 249.71 mg g^−1^ from the BET isotherm model. Since the isotherm and kinetic experiments are not conducted necessarily under optimal conditions, we employed the numerical optimization function in the design expert software to develop scenarios giving the maximum attainable adsorption capacities in the design space. When the optimization was run based on some presumptions, 22 scenarios were defined giving adsorption capacities from 453.84 to 516.02 mg g^−1^.

To compare our prepared adsorbent with previous ones used for AR14 removal from aqueous solutions in terms of adsorption capacity, a systematic search was conducted in Scopus and ScienceDirect with no time limit. The search used a combination of keywords including adsorption, acid red 14, and AR14 in titles, abstracts, and keywords. This strategy led to the retrieval of 43 original articles from Scopus and 63 from ScienceDirect. However, 76 articles were removed (including 60 irrelevant, 14 duplicated, 2 non-English, and 3 not full-text accessible articles). The remaining 27 articles were reviewed in full text to extract the required data including maximum monolayer adsorption capacities from the Langmuir isotherm model. This led to the removal of three extra studies because they did not survey equilibrium data bay the Langmuir model. A summary of the results extracted from the remaining 24 studies is presented in Table S4 in the supplementary file, as compared to the current study results. The retrieved studies were published from 2004 to 2019 and used a variety of adsorbents from natural to synthetic adsorbents. The calculated *q*_*m*_ values based on the Langmuir isotherm ranged from 3.12 to 1940 mg g^−1^, indicating a huge heterogeneity in the results.

### 3.7 Thermodynamic studies

The equations and procedures used for calculating the thermodynamic parameters are presented in Table S5 in the supplemental file. Table 5 summarizes thermodynamic equilibrium constant, *K*_*L*_, and Gibbs free energy changes, *ΔG*^*0*^, at various temperatures.

**Table 5:** Thermodynamic parameters obtained at various temperatures for the removal of AR14 using iron-modified activated carbon (solution pH 4.50, adsorbent dose 0.25 g L^−1^, contact time 40.0 min, initial dye concentration 50–150 mg L^−1^, and solution temperature 278–338 K).

T (K)	K_L_	ΔG^0^ (kJ mol^−1^)	Adj. R^2^ of *q*_*e*_ versus *C*_*e*_ plot
278	7688995	−36.65	0.3811
288	25060831	−40.79	0.9081
298	20070755	−41.66	0.8896
308	6270552	−40.08	0.9105
318	4531989	−40.52	0.9206
328	3039873	−40.71	0.9162
338	2263753	−41.12	0.9404

As can be seen, *ΔG*^*0*^ values were negative at all temperatures, indicating that the adsorption reaction occurred spontaneously. The enthalpy changes of adsorption (*ΔH*^*0*^, kJ mol^−1^) and entropy changes of adsorption (*ΔS*^*0*^, J mol^−1^ K^−1^) were the other thermodynamic parameters calculated in this study. The *ΔH*^*0*^ and *ΔS*^*0*^ values were quantified as −41.86 kJ mol^−1^ and −3.34 J mol^−1^ K^−1^, respectively. The negative *ΔH*^*0*^ value indicates that the adsorption reaction of the dye onto the adsorbent was exothermic, which is supported by the increasing removal rate with decreasing temperature.

## 4 Conclusion

This study was designed to assess the efficacy of activated carbon derived from pistachio shells as agro-waste for dye removal from aqueous solutions. A mesoporous adsorbent was prepared in this study with a surface area of 811.57 m^2^ g^−1^ and a pore volume of 0.654 cm^3^ g^−1^. Iron modification even enhanced the characteristics of activated carbon (surface area by 33.3% and pore volume by 64.1%). The experimental results on acid red-14 adsorption supported the adequacy of iron-modified activated carbon (removal rate up to 99.99%; capacity up to 516 mg g^−1^). Therefore, iron-modified activated carbon derived from pistachio shells could be cost-effective for the treatment of industrial wastewater containing dyes.

## Declarations

### Author contribution statement

Fateme Barjasteh-Askari: Conceived and designed the experiments; Analyzed and interpreted the data; Wrote the paper.

Mojtaba Davoudi: Conceived and designed the experiments; Wrote the paper.

Maryam Dolatabadi: Performed the experiments.

Saeid Ahmadzadeh: Analyzed and interpreted the data.

### Funding statement

This work was supported by the Deputy of Education and Research, Torbat Heydariyeh University of Medical Sciences (Project No. A-10-1306-4).

### Data availability statement

Data will be made available on request.

### Declaration of interests statement

The authors declare no conflict of interest.

### Additional information

No additional information is available for this paper.

## Appendix A Supplementary data

Supplementary file.docx Supplementary file.docx
