# Supplementary material for: Iron-modified activated carbon derived from agro-waste for enhanced dye removal from aqueous solutions
Source: Heliyon. 2021 May 29;7(6):e07191. doi: 10.1016/j.heliyon.2021.e07191 (PMC8188370; doi:10.1016/j.heliyon.2021.e07191)
Supplement: Supplementary file.docx [file mmc1.docx]

**Supplementary materials**

Table S1. Experimental results of AR14 removal based on coded and real values of factors in CCD matrix

| Run order | Coded values | | | | Real values | | | | AR14 removal (%) |
| --- | --- | --- | --- | --- | --- | --- | --- | --- | --- |
|  | X_1_ | X_2_ | X_3_ | X_4_ | A  (mg L^‒1^) | B | C  (g L^‒1^) | D  (min) |  |
| 1 | -1 | -1 | -1 | -1 | 52.5 | 4.75 | 0.15 | 18.75 | 76.58 |
| 2 | 1 | -1 | -1 | -1 | 117.5 | 4.75 | 0.15 | 18.75 | 53.82 |
| 3 | -1 | 1 | -1 | -1 | 52.5 | 8.25 | 0.15 | 18.75 | 43.48 |
| 4 | 1 | 1 | -1 | -1 | 117.5 | 8.25 | 0.15 | 18.75 | 37.92 |
| 5 | -1 | -1 | 1 | -1 | 52.5 | 4.75 | 0.25 | 18.75 | 88.09 |
| 6 | 1 | -1 | 1 | -1 | 117.5 | 4.75 | 0.25 | 18.75 | 59.85 |
| 7 | -1 | 1 | 1 | -1 | 52.5 | 8.25 | 0.25 | 18.75 | 76.56 |
| 8 | 1 | 1 | 1 | -1 | 117.5 | 8.25 | 0.25 | 18.75 | 51.21 |
| 9 | -1 | -1 | -1 | 1 | 52.5 | 4.75 | 0.15 | 46.25 | 83.74 |
| 10 | 1 | -1 | -1 | 1 | 117.5 | 4.75 | 0.15 | 46.25 | 59.07 |
| 11 | -1 | 1 | -1 | 1 | 52.5 | 8.25 | 0.15 | 46.25 | 68.05 |
| 12 | 1 | 1 | -1 | 1 | 117.5 | 8.25 | 0.15 | 46.25 | 40.03 |
| 13 | -1 | -1 | 1 | 1 | 52.5 | 4.75 | 0.25 | 46.25 | 97.16 |
| 14 | 1 | -1 | 1 | 1 | 117.5 | 4.75 | 0.25 | 46.25 | 74.20 |
| 15 | -1 | 1 | 1 | 1 | 52.5 | 8.25 | 0.25 | 46.25 | 94.52 |
| 16 | 1 | 1 | 1 | 1 | 117.5 | 8.25 | 0.25 | 46.25 | 54.60 |
| 17 | -2 | 0 | 0 | 0 | 20 | 6.5 | 0.20 | 32.5 | 99.99 |
| 18 | 2 | 0 | 0 | 0 | 150 | 6.5 | 0.20 | 32.5 | 62.71 |
| 19 | 0 | -2 | 0 | 0 | 85 | 3 | 0.20 | 32.5 | 76.83 |
| 20 | 0 | 2 | 0 | 0 | 85 | 10 | 0.20 | 32.5 | 39.48 |
| 21 | 0 | 0 | -2 | 0 | 85 | 6.5 | 0.10 | 32.5 | 35.34 |
| 22 | 0 | 0 | 2 | 0 | 85 | 6.5 | 0.30 | 32.5 | 79.20 |
| 23 | 0 | 0 | 0 | -2 | 85 | 6.5 | 0.20 | 5 | 50.59 |
| 24 | 0 | 0 | 0 | 2 | 85 | 6.5 | 0.20 | 60 | 78.72 |
| 25 | 0 | 0 | 0 | 0 | 85 | 6.5 | 0.20 | 32.5 | 84.40 |
| 26 | 0 | 0 | 0 | 0 | 85 | 6.5 | 0.20 | 32.5 | 83.57 |
| 27 | 0 | 0 | 0 | 0 | 85 | 6.5 | 0.20 | 32.5 | 85.11 |
| 28 | 0 | 0 | 0 | 0 | 85 | 6.5 | 0.20 | 32.5 | 85.73 |
| 29 | 0 | 0 | 0 | 0 | 85 | 6.5 | 0.20 | 32.5 | 84.50 |
| 30 | 0 | 0 | 0 | 0 | 85 | 6.5 | 0.20 | 32.5 | 82.09 |

A: Initial dye concentration; B: Solution pH; C: Adsorbent dose; D: Contact time

Table S2. The kinetics models used in the study

| Model | Equation | Parameters description |
| --- | --- | --- |
| Pseudo-first order^1^ | $q_{t}=q_{e}\left( 1-e^{{-k}_{1}t} \right)$ | *q_t_* the amount adsorbed (mg g^-1^) at time *t* (min); *q_e_* the amount adsorbed at equilibrium (mg g^-1^); *k_1_* the pseudo-first-order kinetic constant (min^-1^) |
| Pseudo-second order^2^ | $q_{t}=q_{e}\frac{q_{e}k_{2}t}{1+q_{e}k_{2}t}$ | *q_t_* the amount adsorbed (mg g^-1^) at time *t* (min); *q_e_* the amount adsorbed at equilibrium (mg g^-1^); *k_2_* the pseudo-second-order kinetic constant (g mg^-1^ min^-1^) |

^1^ The pseudo-first-order kinetic model describes the adsorption process in proportion to the first power of the number of unoccupied binding sites of the adsorbent, i.e. sorption capacity (Mitić-Stojanović et al., 2012). In this model, it is assumed that the film diffusion step controls the sorption process. This kinetic model shows the irreversible equilibrium state between liquid and solid phases (Zhang et al., 2015). It is well known that this equation best describes the initial steps of the sorption process when the rate of sorption is high and mass transfer controls the process (Mitić-Stojanović et al., 2012). ^2^ The pseudo-second-order kinetic model is in proportion to the square of the number of uncovered binding sites of the sorbent (Mitić-Stojanović et al., 2012). Unlike the former kinetic model, the pseudo-second-order model covers the whole sorption process and considers the three steps including external film diffusion, sorption, and internal particle diffusion (Zhao et al., 2014). Thus, it is more likely to describe the whole process of the sorption until equilibrium.

Table S3. The isotherm models used in the study

| Model | Equation | Parameters description |
| --- | --- | --- |
| Langmuir^1^ | $q_{e}=\frac{q_{m}bC_{e}}{1+bC_{e}}$ | *q_e_* the amount adsorbed at equilibrium (mg g^-1^); *q_m_* the maximum monolayer adsorption capacity (mg g^-1^); *C_e_* the equilibrium concentration in the solution (mg L^-1^); *b* the Langmuir equilibrium constant related to the heat of adsorption (L mg^-1^) |
| Freundlich^2^ | $q_{e}=K_{f}C_{e}^{\frac{1}{n}}$ | *q_e_* the amount adsorbed at equilibrium (mg g^-1^); *C_e_* the equilibrium concentration in the solution (mg L^-1^); *K_f_* ((mg g^-1^) (L mg^-1^)^1/n^) Freundlich constant related to adsorption capacity; *n* Freundlich constant related to adoption intensity |
| Jovanovic^3^ | $q_{e}=q_{m}(1-e^{K_{j}C_{e}})$ | *q_e_* the amount adsorbed at equilibrium (mg g^-1^); *q_m_* the maximum adsorption capacity (mg g^-1^); *C_e_* the equilibrium concentration in the solution (mg L^-1^); *K_j_* is the Jovanovic isotherm constant (L mg^-1^) |
| Redlich-Peterson^4^ | $qe=\frac{AC_{e}}{1+BC_{e}^{g}}$ | *q_e_* the amount adsorbed at equilibrium (mg g^-1^); *C_e_* the equilibrium concentration in the solution (mg L^-1^); *A* (L g^-1^) and *B* ((mg L^-1^)^-g^) the Redlich-Peterson isotherm constants; *g* the Redlich-Peterson isotherm exponent |
| BET^5^ | $q_{e}=\frac{q_{s}C_{BET}C_{e}}{(\left( C_{s}-C_{e} \right)\left[ 1+\left( C_{BET}-1 \right)\left( \frac{C_{e}}{C_{s}} \right) \right])}$ | *q_e_* the amount adsorbed at equilibrium (mg g^-1^); *C_e_* the equilibrium concentration in the solution (mg L^-1^); *q_s_* the theoretical isotherm saturation capacity (mg g^-1^); *C_BET_* the BET adsorption isotherm constant related to the energy of surface interaction (L mg^-1^); *C_s_* the adsorbate monolayer saturation concentration (mg L^-1^) |

^1^ The Langmuir isotherm as a two-parameter model explains the monolayer adsorption on the homogenous surfaces of an adsorbent (Gholizadeh et al., 2013). It also assumes identical sorption sites with finite numbers and no interaction between adsorbed solutes (Bazrafshan et al., 2015). ^2^ The Freundlich model explains multilayer adsorption onto the heterogeneous surface of an adsorbent (Yoosefian et al., 2017). ^3^ The Jovanovic isotherm is used to describe monolayer localized adsorption of molecules with no lateral interactions (Rahmani et al., 2018). Technically, this model is similar to the Langmuir model except that the former allows for the surface binding vibrations of adsorbate (Farouq and Yousef, 2015). ^4^ The Redlich-Peterson isotherm is a model with three parameters applicable to an extensive adsorbate concentration range in homogenous or heterogeneous adsorption systems (Rahmani et al., 2018). Thus, it is inferred that the Langmuir model is a special case of the Redlich-Peterson model when the exponent equals unity. ^5^ The BET isotherm model is a three-parameter model to describe the interaction between adsorbate and adsorbent. It is a modified form of the Langmuir model to explain multilayer adsorption (Saghapour et al., 2013).

Table S4. The results of a systematic literature search for studies assessing adsorption capacities for AR14 using calculated by the Langmuir model

|  | **Author, year** | **Origin country** | **Adsorbent** | **Time** | **Concentration mg/l** | **T (°C)** | **q_m_ (mg/g)** | **R^2^** |
| --- | --- | --- | --- | --- | --- | --- | --- | --- |
| 1 | Chiou et al. (2004) | Taiwan | Crosslinked chitosan beads | 5 days | UN | 30 | 1940 | 0.9893 |
| 2 | Arami et al. (2006) | Iran | Soy meal hull | 24 h | 50-150 | 20 | 109.89 | 0.9681 |
| 3 | Arami et al. (2008) | Iran | Eggshell membrane | 2 h | UN | 20 | 513.98 | 0.826 |
| 4 | Gao et al. (2011) | China | Treated okara | 24 h | 100-500 | 20 | 217.39 | 0.9968 |
| 5 | Mahmoodi et al. (2011) | Iran | Amine-functionalized silica nanoparticle | 60 min | 50 | 25 | 434 | 0.987 |
| 6 | Haji and Mahmoodi (2012) | Iran | Soy meal hull activated carbon | 60 min | 50 | 25 | 21.37 | 0.998 |
| 7 | Hayati and Mahmoodi (2012) | Iran | Alkaline modified activated carbon | UN | UN | 25 | 9.17 | 0.8 |
| 8 | Samarghandi et al. (2012a) | Iran | Treated pumice | 24 h | 50-120 | 20 | 3.12 | 0.9699 |
| 9 | Samarghandi et al. (2012b) | Iran | Pumice stone | UN | UN | UN | 58.82 | 0.977 |
| 10 | Yazdani et al. (2012) | Iran | Surfactant-modified feldspar | 90 min | 10-250 | 25 | 3.98 | 0.99 |
| 11 | Mahmoodi and Najafi (2012) | Iran | High aminefunctionalized titania/silica nano-hybrid | 60 min | 50 | 25 | 312.5 | 0.989 |
| 12 | Mahmoodi (2013) | Iran | Amine-functionalized magnetic ferrite nanoparticle | 60 min | 50 | 25 | 147.06 | 0.999 |
| 13 | FARAH and ELGENDY (2013) | Egypt | Waste biomass of saccharomyces cerevisiae | 300 min | 30-4000 | 25 | 499.37 | 0.989 |
| 14 | Mahmoodi et al. (2014) | Iran | Zinc aluminum hydroxide | UN | UN | 25 | 84 | 0.999 |
| 15 | Khodam et al. (2015) | Iran | Nicoal-layered double hydroxide/multi-walled carbon nanotubes | UN | 50 | 25 | 196.08 | 0.9946 |
| 16 | Hosseinabadi-Farahani et al. (2015) | Iran | Amine functionalized graphene oxide nanosheet | UN | UN | 25 | 64 | 0.8637 |
| 17 | Ghani et al. (2016) | Iran | Novel cross-linked superfine alginate-based nanofibers | UN | UN | room | 17.95 | 0.9901 |
| 18 | Shojaat et al. (2017) | Iran | MnFe2O4 /Calcium alginate | 3 h | 10--75 | 30 | 4.77 | 0.978 |
| 19 | Najafi et al. (2016) | Iran | Tomato seeds | UN | UN | 30 | 125 | 0.986 |
| 20 | Ghasempour et al. (2017) | Iran | Poly(propylene imine)-modified graphene oxide | 30 min | 50-125 | 25 | 434.78 | 0.99 |
| 21 | Shirzad-Siboni et al. (2018) | Iran | Scallop shell coated with Fe3O4 | 72 h | 30 | 25 | 172.41 | 0.9906 |
| 22 | Bazrchi et al. (2018) | Iran | Poly(styrene-alternative-maleic anhydride) (PSMA) polymer | UN | UN | 25 | 5.12 | 0.993 |
| 23 | Wang et al. (2019) | China | CeO_2_·*x*H_2_O | 30 min | 100.4-753 | room | 540 | UN |
| 24 | Current study | Iran | Pistachio shells activated carbon | 40 min | 30-150 | 22 | 328.44 | 0.92 |

Table S5. The equations used for calculating thermodynamic parameters in this study

| Equation | Parameter description | Procedure |
| --- | --- | --- |
| ${\Delta G}^{0}=-RTlnK_{L}$ | *ΔG^0^* Gibbs free energy change, *R* the universal gas constant (8.314 J mol^-1^ K^-1^), *T* the absolute temperature (K), *K_L_* the thermodynamic equilibrium constant (dimensionless) | *K_L_* was calculated at various temperatures (278-338 K) and thus gave various *ΔG^0^* values. |
| $K_{L}=55.5b$ | *b* the Langmuir constant (L mol^-1^) | Parameter *b* was obtained from the non-linear plot of *q_e_* (mmol g^-1^) versus *C_e_* (mol L^-1^); The value of 55.5 is the mole numbers of water per liter of solution (mol L^-1^), which is introduced to have dimensionless *K_L_* values |
| $lnK_{L}=-\frac{\Delta H^{0}}{RT}+\frac{\Delta S^{0}}{R}$ | *ΔH^0^* enthalpy changes of adsorption (kJ mol^-1^), *ΔS^0^* entropy changes of adsorption (J mol^-1^ K^-1^) | The plot of *lnK_L_* versus *1/T* gave a straight line, in which the slope and intercept are used to calculate *ΔH^0^* and *ΔS^0^*, respectively. The plot in this study was drawn using *K_L_* values in Table 6 except, for *K_L_* at 278 K due to its poor coefficient of determination. The obtained plot gave a line with a slope of 5034.8 and an intercept of -0.4023 (R^2^=0.9548). |

Fig. S1. The results of data fitting to kinetic models for the removal of AR14 using iron-modified activated carbon; The experimental conditions for conducting kinetic studies included solution pH of 4.50, contact time of 0-40.00 min, adsorbent dose of 0.25 g L^-1^, and initial dye concentration of 100.00 mg L^-1^.

Fig. S2. The results of data fitting to isotherm models for the removal of AR14 using iron-modified activated carbon; The experimental conditions for conducting isotherm studies included solution pH of 4.50, equilibrium contact time of 40.00 min, adsorbent dose of 0.25 g L^-1^, and initial dye concentration of 30 to 150 mg L^-1^.

**References:**

ARAMI, M., LIMAEE, N. Y. & MAHMOODI, N. M. 2008. Evaluation of the adsorption kinetics and equilibrium for the potential removal of acid dyes using a biosorbent. *Chemical Engineering Journal,* 139**,** 2-10.

ARAMI, M., LIMAEE, N. Y., MAHMOODI, N. M. & TABRIZI, N. S. 2006. Equilibrium and kinetics studies for the adsorption of direct and acid dyes from aqueous solution by soy meal hull. *Journal of Hazardous Materials,* 135**,** 171-179.

BAZRAFSHAN, E., KORD MOSTAFAPOUR, F., RAHDAR, S. & MAHVI, A. H. 2015. Equilibrium and thermodynamics studies for decolorization of Reactive Black 5 (RB5) by adsorption onto MWCNTs. *Desalination and Water Treatment,* 54**,** 2241-2251.

BAZRCHI, S., BAHRAM, M. & NOURI, S. 2018. Equilibrium and kinetic studies on the removal of acid red-14 from aqueous solutions using PSMA. *Iranian Journal of Science and Technology, Transactions A: Science,* 42**,** 203-208.

CHIOU, M.-S., HO, P.-Y. & LI, H.-Y. 2004. Adsorption of anionic dyes in acid solutions using chemically cross-linked chitosan beads. *Dyes and pigments,* 60**,** 69-84.

FARAH, J. Y. & ELGENDY, N. 2013. Performance, kinetics and equilibrium in biosorption of anionic dye Acid Red 14 by the waste biomass of Saccharomyces cerevisiae as a low-cost biosorbent. *Turkish Journal of Engineering and Environmental Sciences,* 37**,** 146-161.

FAROUQ, R. & YOUSEF, N. 2015. Equilibrium and kinetics studies of adsorption of copper (II) ions on natural biosorbent. *International Journal of Chemical Engineering and Applications,* 6**,** 319.

GAO, J.-F., WANG, J.-H., YANG, C., WANG, S.-Y. & PENG, Y.-Z. 2011. Binary biosorption of Acid Red 14 and Reactive Red 15 onto acid treated okara: Simultaneous spectrophotometric determination of two dyes using partial least squares regression. *Chemical engineering journal,* 171**,** 967-975.

GHANI, M., REZAEI, B., GHARE AGHAJI, A. & ARAMI, M. 2016. Novel Cross‐linked Superfine Alginate‐Based Nanofibers: Fabrication, Characterization, and Their Use in the Adsorption of Cationic and Anionic Dyes. *Advances in Polymer Technology,* 35**,** 428-438.

GHASEMPOUR, A., PAJOOTAN, E., BAHRAMI, H. & ARAMI, M. 2017. Introduction of amine terminated dendritic structure to graphene oxide using Poly (propylene Imine) dendrimer to evaluate its organic contaminant removal. *Journal of the Taiwan Institute of Chemical Engineers,* 71**,** 285-297.

GHOLIZADEH, A., KERMANI, M., GHOLAMI, M. & FARZADKIA, M. 2013. Kinetic and isotherm studies of adsorption and biosorption processes in the removal of phenolic compounds from aqueous solutions: comparative study. *Journal of environmental health science and engineering,* 11**,** 29.

HAJI, A. & MAHMOODI, N. M. 2012. Soy meal hull activated carbon: preparation, characterization and dye adsorption properties. *Desalination and Water Treatment,* 44**,** 237-244.

HAYATI, B. & MAHMOODI, N. M. 2012. Modification of activated carbon by the alkaline treatment to remove the dyes from wastewater: mechanism, isotherm and kinetic. *Desalination and Water Treatment,* 47**,** 322-333.

HOSSEINABADI-FARAHANI, Z., MAHMOODI, N. M. & HOSSEINI-MONFARED, H. 2015. Preparation of surface functionalized graphene oxide nanosheet and its multicomponent dye removal ability from wastewater. *Fibers and Polymers,* 16**,** 1035-1047.

KHODAM, F., REZVANI, Z. & AMANI-GHADIM, A. R. 2015. Enhanced adsorption of Acid Red 14 by co-assembled LDH/MWCNTs nanohybrid: Optimization, kinetic and isotherm. *Journal of Industrial and Engineering Chemistry,* 21**,** 1286-1294.

MAHMOODI, N. M. 2013. Synthesis of amine-functionalized magnetic ferrite nanoparticle and its dye removal ability. *Journal of Environmental Engineering,* 139**,** 1382-1390.

MAHMOODI, N. M., KHORRAMFAR, S. & NAJAFI, F. 2011. Amine-functionalized silica nanoparticle: Preparation, characterization and anionic dye removal ability. *Desalination,* 279**,** 61-68.

MAHMOODI, N. M., MASROURI, O. & ARABI, A. M. 2014. Synthesis of porous adsorbent using microwave assisted combustion method and dye removal. *Journal of Alloys and Compounds,* 602**,** 210-220.

MAHMOODI, N. M. & NAJAFI, F. 2012. Synthesis, amine functionalization and dye removal ability of titania/silica nano-hybrid. *Microporous and Mesoporous Materials,* 156**,** 153-160.

MITIĆ-STOJANOVIĆ, D.-L., BOJIĆ, D., MITROVIĆ, J., ANĐELKOVIĆ, T., RADOVIĆ, M. & BOJIĆ, A. L. 2012. Equilibrium and kinetic studies of Pb (II), Cd (II) and Zn (II) sorption by Lagenaria vulgaris shell. *Chemical Industry and Chemical Engineering Quarterly/CICEQ,* 18**,** 563-576.

NAJAFI, H., PAJOOTAN, E., EBRAHIMI, A. & ARAMI, M. 2016. The potential application of tomato seeds as low-cost industrial waste in the adsorption of organic dye molecules from colored effluents. *Desalination and Water Treatment,* 57**,** 15026-15036.

RAHMANI, A., NAZEMI, F., BARJASTEH-ASKARI, F. & DAVOUDI, M. 2018. Preparation, Characterization, and Application of Silica Aerogel for Adsorption of Phenol: An In-Depth Isotherm Study. *Health Scope,* 7.

SAGHAPOUR, Y., AGHAIE, M. & ZARE, K. 2013. Thermodynamic study of lead ion removal by adsorption onto nanographene sheets.

SAMARGHANDI, M. R., ZARRABI, M., AMRANE, A., SAFARI, G. H. & BASHIRI, S. 2012a. Application of acidic treated pumice as an adsorbent for the removal of azo dye from aqueous solutions: kinetic, equilibrium and thermodynamic studies. *Iranian journal of environmental health science & engineering,* 9**,** 9.

SAMARGHANDI, M. R., ZARRABI, M., NOORI SEPEHR, M., PANAHI, R. & FOROGHI, M. 2012b. Removal of Acid Red 14 by pumice stone as a low cost adsorbent: kinetic and equilibrium study. *Iranian journal of chemistry and chemical engineering,* 31**,** 19-27.

SHIRZAD-SIBONI, M., MOHAGHEGHIAN, A., VAHIDI-KOLUR, R., POURMOHSENI, M. & YANG, J.-K. 2018. Preparation and characterization of Scallop shell coated with Fe3O4 nanoparticles for the removal of azo dye: Kinetic, equilibrium and thermodynamic studies. *Indian Journal of Chemical Technology (IJCT),* 25**,** 40-50.

SHOJAAT, R., KARIMI, A., SAADATJOO, N. & ABER, S. 2017. Dye removal from artificial wastewater using heterogeneous bio-fenton system. *Chemical Industry and Chemical Engineering Quarterly,* 23**,** 447-456.

WANG, H., ZHONG, Y., YU, H., APREA, P. & HAO, S. 2019. High-efficiency adsorption for acid dyes over CeO2· xH2O synthesized by a facile method. *Journal of Alloys and Compounds,* 776**,** 96-104.

YAZDANI, M., MAHMOODI, N. M., ARAMI, M. & BAHRAMI, H. 2012. Surfactant‐modified feldspar: Isotherm, kinetic, and thermodynamic of binary system dye removal. *Journal of applied polymer science,* 126**,** 340-349.

YOOSEFIAN, M., AHMADZADEH, S., AGHASI, M. & DOLATABADI, M. 2017. Optimization of electrocoagulation process for efficient removal of ciprofloxacin antibiotic using iron electrode; kinetic and isotherm studies of adsorption. *Journal of Molecular Liquids,* 225**,** 544-553.

ZHANG, L., LIU, Y., WANG, S., LIU, B. & PENG, J. 2015. Selective removal of cationic dyes from aqueous solutions by an activated carbon-based multicarboxyl adsorbent. *RSC advances,* 5**,** 99618-99626.

ZHAO, Y., LI, J., ZHAO, L., ZHANG, S., HUANG, Y., WU, X. & WANG, X. 2014. Synthesis of amidoxime-functionalized Fe3O4@ SiO2 core–shell magnetic microspheres for highly efficient sorption of U (VI). *Chemical Engineering Journal,* 235**,** 275-283.
